# Impact of heart failure and other comorbidities on mortality in patients with chronic obstructive pulmonary disease: a register-based, prospective cohort study

**DOI:** 10.1186/s12875-018-0865-8

**Published:** 2018-11-24

**Authors:** Elzbieta Kaszuba, Håkan Odeberg, Lennart Råstam, Anders Halling

**Affiliations:** 1 Samaritens Primary Health Care Centre, 374 80 Karlshamn, Sweden; 2 Department of Clinical Sciences in Malmö General Practice, Center for Primary Health Care Research, Lund University, 205 02, Malmö, Sweden

**Keywords:** Chronic obstructive pulmonary disease, Heart failure, Mortality, Age, Sex, Comorbidity

## Abstract

**Background:**

Multimorbidity has already become common in primary care and will be a challenge in the future. Primary care in Sweden participates to a great extent in the care of patients with two severe, chronic conditions: chronic obstructive pulmonary disease (COPD) and heart failure. Both conditions are characterized by high mortality and often coexist. Age, sex, heart failure and other comorbidities are considered to be the major predictors of mortality in patients with COPD. We aimed to study the impact of heart failure, other comorbidities, age and sex on mortality in patients with COPD.

**Methods:**

A register-based, prospective cohort study conducted in Blekinge County in Sweden with about 150,000 inhabitants. The study population was comprised of people aged ≥35 years. The data about diagnoses of COPD and heart failure came from the 2007 health care register, in which we found 984 individuals with a diagnosis of COPD. Date of death was collected from January 1st, 2008 –August 31st, 2015. The diagnosis-based Adjusted Clinical Groups (ACG) Case-Mix System 7.1 was used to describe comorbidity. Each individual was assigned one of six comorbidity levels called resource utilization bands (RUB) graded from 0 to 5.

**Results:**

Estimated eight year mortality in patients with COPD and coexisting heart failure was seven times higher than in patients with COPD alone - odds ratio 7.06 (95% CI 3.88–12.84). Adjusting for age and male sex resulted in odds ratio 3.75 (95% CI 1.97–7.15). Further adjusting for other comorbidities resulted in odds ratio 3.26 (95% CI 1.70–6.25).

The mortality was strongly associated with the highest comorbidity level – RUB 5 where the odds ratio was 5.19 (95% CI 2.59–10.38).

**Conclusion:**

Heart failure has an important impact on mortality in patients with COPD. The mortality in patients with COPD and coexisting heart failure was strongly associated with age, male sex and other comorbidities. Of those three predictors, only other comorbidities can be influenced. Heart failure and other comorbidities should be recognized early and properly treated in order to improve survival in patients with coexisting COPD and heart failure.

## Background

General practitioners nowadays seldom encounter patients with only one disease in daily practice. With an aging population there is, therefore, an increased likelihood of having two or more chronic diseases simultaneously among this population [1]. Multimorbidity has already become the norm and will be a challenge for future primary care. Our previous study showed that primary care in Sweden participates to a great extent in the care of patients with two severe, chronic conditions: chronic obstructive pulmonary disease (COPD) and heart failure [2]; both conditions are common in the Swedish population [3–5]. Prevalence of COPD is still high despite a decrease in the number of smokers in Sweden [6]. By the year 2030, COPD is predicted to be the third most common cause of death and disability worldwide [7]. High mortality is not caused by respiratory failure, which from the pathophysiological point of view can be considered a natural consequence of COPD due to disturbances in gas exchange [8]. Respiratory failure was a leading cause of death only in patients with advanced COPD and could explain mortality in only a third of these patients [9]. The major independent predictors of mortality in COPD were comorbidity and sex [10–12]. Heart failure occurs with high prevalence in patients with COPD [13]. Although the strategies in management of heart failure have improved during recent decades, the prognosis remains poor [14–16]. Comorbidities, age and sex are independent indicators for the prognosis in heart failure [17, 18]. Despite an increased understanding that COPD and heart failure often coexist [19], both have mostly been studied separately in prior studies. It is a matter of course that the more disorders an individual has the worse the outcome will be. However, it is not clear if one plus one makes two in medical practice. The aim of this study was to find how much coexisting heart failure increases the probability of death in patients with COPD and to describe the impact of age, sex and other comorbidities on mortality in patients with coexisting COPD and heart failure. Thereby we wanted to find what coexisting heart failure and other comorbidities mean for the survival of an individual with COPD, which is of special importance in primary care where the patient is the main focus.

## Methods

This was a register-based, prospective, observational study. The study was conducted in Blekinge County in Sweden, which has about 150,000 inhabitants. The study population was comprised of people aged ≥35 years.

Data were obtained from the Blekinge County council health care register; a register that collects data concerning diagnosis at each consultation in all public and private health care units in both primary- and secondary health care. Diagnoses were recorded according to the International Statistical Classification of Diseases and Related Health Problems version 10 (ICD 10). We analyzed data from January 1st till December 31st, 2007.

An individual was identified as having COPD if the diagnosis code J44 was recorded on at least one consultation in primary- or secondary care including hospitalization. The code J44 comprised the following: J44 chronic obstructive lung disease, J44.0 chronic obstructive lung disease with acute infection in lower airways, J44.1 chronic obstructive lung disease with acute exacerbation, unspecified, J44.8 other specified chronic obstructive lung disease including chronic bronchitis with emphysema. The register did not contain information on the severity of COPD.

Coexisting heart failure was identified if the diagnosis code I50 was recorded.

The code I50 comprised: I50.0- chronic heart failure including congestive heart failure, right heart failure secondary to left heart failure, I50.1- left ventricular failure with or without lung oedema and asthma cardiale, I50.9 heart failure, unspecified. Occurrence of other diagnoses in medical records is referred to as other comorbidities.

The diagnosis-based Adjusted Clinical Groups (ACG) Case-Mix System 7.1 was used to measure other comorbidities [20, 21]. Each individual was assigned one of six comorbidity levels called resource utilization bands (RUB) graded from 0 to 5, where 5 means very high morbidity and need for care.

The date of death was collected from January 1st, 2008 –August 31st, 2015 and was obtained from the Blekinge County council. Individuals who left the Blekinge County during the observation time were excluded from the study.

### Statistics

Data were analyzed in the STATA version 13 (Stata Corporation, Texas, USA).

Distribution of categorical variables was presented as numbers. Distribution of continuous variables was presented as a mean and 95% Confidence Interval (CI).

Univariate analysis was used to calculate odds ratios of mortality for different variables. Logistic regression was used to calculate adjusted odds ratios step-by-step. Thereafter, multiple logistic regression was performed in order to adjust the estimated odds ratio for the influence of age, sex and other comorbidities.

## Results

The study population consisted of 91,227 individuals aged ≥35 years of which 51.07% were female and 48.93% were male. The diagnosis of COPD was registered in 1011 individuals of which 27 (2.67%) moved from the county during the observation time and were thus excluded from the study. The final analyses were made based on data from 984 individuals; 53% female and 47% male. The mean age in patients with the diagnosis of COPD was 71.1 (95% CI 70.4–71.8). The mean age for women was 69.4 (95% CI 68.5–70.4) and for men 72.9 (95% CI 72.0–73.8).

Comorbidity levels described by RUB were distributed as follows: RUB 3–71.14% (*n* = 700), RUB 4–21.65% (*n* = 213), RUB 5–7.22% (*n* = 71).

We found the diagnosis of heart failure in 10.06% (*n* = 99) of patients with the diagnosis of COPD.

Univariate analysis resulted in an odds ratio of mortality in patients with COPD and coexisting heart failure of 7.06 (95% CI 3.88–12.84). The highest level of other comorbidities was the next important variable that influenced mortality (Table 1).

**Table 1:** Odds ratios of mortality in patients with COPD – univariate analyses of different variables used

OR (95%CI)	OR (95%CI)
Heart failure	7.06 (3.88–12.84)
Age	1.13 (1.11–1.14)
Male sex	1.78 (1.38–2.30)
RUB 4	2.03 (1.48–2.78)
RUB 5	4.78 (2.61–8.74)

Adjusting for age resulted in odds ratio 3.76 (95% CI 1.98–7.16) and for age and male sex in odds ratio 3.75 (95% CI 1.79–3.26 (1.70–6.25). Further adjusting for other comorbidities resulted in odds ratio 3.26 (95% CI 1.70–6.25). Adjusted odds ratios are presented in Table 2.

**Table 2:** Odds ratios (OR) of mortality in heart failure in patients with COPD adjusted for different variables used

Variables	OR (95%CI)
Heart failure	7.06 (3.88–12.84)
Heart failure, age	3.76 (1.98–7.16)
Heart failure, age, male sex	3.75 (1.97–7.15)
Heart failure, age, male sex, other comorbidities	3.26 (1.70–6.25)

Multiple logistic regression showed that the mortality was associated with age and male sex but the strongest risk factor for mortality was the highest comorbidity level – RUB 5 where the odds ratio was 5.19 (95% CI 2.59–10.38) (Table 3). The most common comorbid diagnoses in our material were: hypertension (20%), ischaemic heart disease (12.7%), diabetes mellitus (12.6%) and atrial fibrillation (7.9%). The odds ratio was the highest for RUB 5,even after adjusting for those comorbid diagnoses (Table 4).

**Table 3:** Importance of different variables for mortality in patients with COPD. Odds ratios (OR) were adjusted for all other variables

Variables	Adjusted OR (95%CI)
Heart failure	3.26 (1.70–6.25)
Age	1.12 (1.10–1.14)
Male sex	1.37 (1.02–1.85)
RUB 4	1.82 (1.26–2.65)
RUB 5	5.19 (2.59–10.38)

**Table 4.** Importance of different variables including the most common comorbid diagnoses for mortality in patients with COPD. Odds ratios (OR) were adjusted for all other variable

Variables	Adjusted OR (95% CI)
Heart failure	3.79 (1.94 -7.43)
Hypertension	0.56 (0.39 - 0.81)
Ischemic heart disease	0.94 (0.58 - 1.51)
Diabetes mellitus	1.76 (1.12 - 2.78)
Atrial fibrillation	2.07 (1.08 - 3.96)
Age	1.08 (1.07-1.10)
Male sex	1.56 (1.16 - 2.09)
RUB 4	1.82 (1.26 - 2.62)
RUB 5	5.22 (2.64 -10.32)

## Discussion

We found that the odds of eight year mortality in patients with COPD and coexisting heart failure were seven times higher than in patients with COPD alone. Other comorbidities were found to be a very important factor affecting mortality in patients with COPD and coexisting heart failure.

Heart failure was previously reported as the most common comorbidity noted in deceased patients that had been hospitalized with COPD exacerbation [22]. The mortality risk in patients with COPD in primary care was more than doubled when heart failure occurred as a comorbidity [23]. Our study confirmed that heart failure considerably increases the probability of death in patients with COPD in the general population. This is not surprising when taking into consideration that COPD is associated with an increased risk of cardiovascular diseases [24] due to smoking as a common risk factor and systemic inflammation, oxidative and physiologic stress and vascular dysfunction as a common mechanism [25]. Age and male sex were associated with higher mortality in patients with COPD and coexisting heart failure, as expected when taking into consideration data on heart failure [17] and COPD alone [22]. A wide spectrum of comorbidities was previously reported as factors associated with mortality for patients with heart failure and COPD alone. The comorbidities with the highest impact on mortality in patients with heart failure were: COPD, stroke, renal disease, anemia, diabetes [15] and for patients with COPD: respiratory failure, pneumonia, heart failure, ischaemic heart disease, hypertension and lung cancer [26]. The importance of comorbidity for mortality was shown in a Swedish study in patients with severe COPD treated with long-term oxygen therapy [27]. We showed the same effect of comorbidity in a whole population of patients with COPD. The main aim in our study, however, was not to find out if but how much heart failure, age, sex and other comorbidities influence mortality in patients with COPD. It is known that prognosis in heart failure is poorer than in COPD [28, 29] and higher mortality in patients with coexisting COPD and heart failure was expected. By adjusting odds ratios for heart failure we wanted to show importance of other comorbidities; early identifying and proper management of those may decrease mortality.

Other comorbidity in our study was calculated as an individual measure using diagnoses analyzed with the ACG Case Mix system. The severity and chronicity of the different diagnoses in an individual led to a higher level of comorbidity (RUB). RUB is a categorization of the individual patient’s morbidity and describes the sum of all comorbidities in an individual. We showed that the higher the level of comorbidity, the higher the odds ratio of mortality even when presence of heart failure was adjusted for. Odds ratio of mortality adjusted for age and male sex in the group with the highest level of other comorbidity was five times higher. Adjusting for the most common comorbidities: hypertension, ischaemic heart disease, diabetes mellitus and atrial fibrillation did not considerably change the importance of RUB as a summarizing measurement of all other comorbidities.

This means that other comorbidities are an important independent predictor of mortality;

this should be recognized when treating patients with COPD and coexisting heart failure.

An interesting finding when adjusting for major comorbidities was that the presence of the diagnosis of hypertension lowered odds ratio of mortality. Hypertension was previously reported as an important comorbid condition which contributed to a higher risk of mortality in patients with COPD [30]. A possible explanation of this finding can be benefits associated with more extensive cardiovascular medication in patients with known hypertension [31]. This would be worth looking into in more detail in further studies.

### Strengths and limitations

Our study was register-based and the primary source of information in the register was the patient’s medical record, which limited baseline characteristics to age and sex. The register collected data concerned diagnosis at each consultation in all public and private care units. The limitations that we had to deal with were completeness of data and validity of the diagnoses. Due to the listing system in primary care in the Blekinge County we could study the whole population, which is a strength of our study. Each inhabitant was either actively or passively listed with a primary care center. Data from both primary- and secondary care were collected in order to increase completeness of data. COPD and heart failure are heterogeneous diagnoses. In order to capture as many cases as possible we used all codes which are available under the main codes for COPD and heart failure according to ICD 10 classification. The limitation of our study is validity of diagnoses of COPD and heart failure in the register. Validating of diagnoses against the medical records at an individual level was not possible for ethical reasons. Previous studies, however, showed that both diagnoses in Swedish registers had an acceptable level of validity for being used in epidemiological research [32, 33]. Other studies showed that data from Swedish registers can be used in estimating prevalence of chronic diseases such as hypertension, ischaemic heart disease, COPD or diabetes [34].

## Conclusion

Coexisting heart failure considerably increases the odds of mortality in patients with COPD.

The mortality in patients with COPD and coexisting heart failure is strongly associated with age, male sex and other comorbidities. Of those three predictors, only other comorbidities can be influenced. It is important to recognize other comorbidities early and to treat them adequately in patients with COPD and coexisting heart failure since they significantly influence survival.

## Abbreviations

ACG: Adjusted clinical groups
CI: Confidence interval
COPD: Chronic obstructive pulmonary disease
RUB: Resource utilization band
